# Pacific Healthy Islands Vision: success factors and challenges faced by health promotion programs

**DOI:** 10.1093/heapro/daac002

**Published:** 2022-06-14

**Authors:** Erik Martin, Wendy Snowdon, Ada Moadsiri, Saula Volavola, Colin Bell

**Affiliations:** School of Medicine, Deakin University, Geelong, Australia; Division of Pacific Technical Support, World Health Organization, Suva, Fiji; Division of Pacific Technical Support, World Health Organization, Suva, Fiji; Division of Pacific Technical Support, World Health Organization, Suva, Fiji; School of Medicine, Deakin University, Geelong, Australia

**Keywords:** healthy settings, community-based intervention, Pacific Islands, health-promoting environments

## Abstract

The World Health Organization’s (WHO) Western Pacific Regional Office developed the biennial Healthy Islands Recognition Awards (HIA) in 2009 to reinforce the Healthy Islands vision and encourage countries to continue to innovate and demonstrate effective and efficient ways of promoting and protecting population health. This research aimed to identify characteristics of and challenges for successful health promotion in the Pacific. The research was undertaken to develop practical guidance for other groups in the Pacific Islands interested in supporting Healthy Islands. We used a qualitative case study to review 2013 and 2015 HIA awardees from eight Pacific Island countries and territories using a set of questions drawn from the HIA application criteria. In 2015–2016, 35 key informant interviews and a review of program documents were undertaken. This was followed by a workshop with representatives from three HIA awardees to further develop recommendations. We reviewed eight programs targeting healthy eating, physical activity, healthy settings and sanitation. Using evidence, careful planning, building capacity, developing partnerships, strengthening and reorientating networks, ensuring accountability and conducting evaluation were keys to the success of healthy islands projects. Considering the local setting and community was perhaps the most crucial theme amongst the programs examined. Challenges included funding and capacity constraints, maintaining commitment and prioritisation, maintaining communication and coordination and technical challenges. Success factors, challenges and recommendations aligned well with mainstream health promotion literature, although some important distinctions exist. Further research is needed to guide successful health promotion practice in the Pacific.

## BACKGROUND

The concept of Healthy Islands was envisioned at the first Pacific Island Ministers of Health meeting, Yanuca Island, Fiji in 1995 in response to emerging health challenges faced by Pacific island countries. The *Yanuca Island Declaration* (WHO, 1995), declared that Healthy Islands should be places where children are nurtured in body and mind; environments invite learning and leisure; people work with age and dignity; ecological balance is a source of pride; and the ocean which sustains us is protected^1^.

Since 1995, this vision has been reaffirmed in numerous Pacific Island Health Ministers meetings, including the *2015 Yanuca Island Declaration on health in Pacific island countries and territories* which celebrated the 20th anniversary of the Healthy Islands vision. Healthy Islands adopts a settings-based approach to health promotion, taking account of ‘the place or social context in which people engage in daily activities in which environmental, organisational, and personal factors interact to affect health and wellbeing’ (WHO, 1998, p. 13). Galea *et al.* (2000) suggested that Healthy Islands are a broad contextual setting, enclosing other contexts and elements, such as cities and schools. The vision is frequently referred to as inspirational by Pacific leaders, taking it beyond settings into the hearts of Pacific people (WHO, 2015). WHO (2015) noted that Healthy Islands, as a brand, is subject to a wide range of interpretations and dynamics; countries use it however it best fits, and the concept evolves over time. It is not surprising then, that the vision is expressed in a plethora of ways, from mobilising people in a single village to clean up waste and remove pools of water to improve sanitation and control malaria, to using media to encourage awareness of a netball program to promote physical activity and reduce the burden of non-communicable diseases (NCDs).

The Healthy Islands Recognition Awards (HIA) program was established by the WHO Regional Office for the Western Pacific in 2009 to reinforce the Healthy Islands vision and to encourage communities and countries to continue to innovate and demonstrate effective and efficient ways of promoting and protecting the health of their populations. Visions can be challenging to implement and in 2013, the WHO published a *Framework of Action for Revitalization of Healthy Islands in the Pacific* to highlight some challenges and carry the Healthy Islands vision forward (WHO, 2013). The WHO Regional Office for the Western Pacific invited applications for the HIA program under ‘best practice’ and ‘best proposal’ categories to recognise both established projects and innovative projects. All applications were evaluated by two independent public health academics from Deakin University with expertise in health promotion and experience working in the Pacific. HIA criteria included originality and innovation, alignment with Healthy Islands vision, alignment with local needs, measurable and achievable improvements in health and quality of life, promotion of healthy environments, local capacity, transferability and the incorporation of a recognition category (which included government support, community-based efforts or partnerships). These were developed after a review of earlier criteria against the principles of the Yanuca Islands Declaration and the Ottawa Charter for Health Promotion (E. de Leeuw & E. Martin, unpublished report). Shortlisted proposals were reviewed by a panel, consisting of the two aforementioned public health academics and a representative from the WHO Regional Office for the Western Pacific for final selection. HIA recognises outstanding work, particularly in community-based actions and efforts, partnership efforts, and governmental support for Healthy Islands initiatives by respective programs and people from diverse backgrounds. Between 2013 and 2015, there were eight awardees from eight different Pacific Island countries or territories. While the awarded programs addressed a range of public health issues at various levels, five of them sought to address chronic disease and three focused on improved sanitation through modifications to the built environment.

Much of the literature on effective health promotion is based in settings that are contextually different from the Pacific Islands and health promotion literature in the Pacific is scarce. Even though many health promotion programs are delivered on the ground, few are fully evaluated or published in the peer-reviewed literature. The aim of this research was to review 2013 and 2015 awarded programs to identify characteristics of success and challenges for health promotion in the Pacific Islands.

## METHODS

A qualitative case study design was utilised, the preferred method when the researcher seeks to investigate in-depth questions on contemporary phenomena where there is little or no control (Yin, 2014). The suitability of the case study approach has been recognised for evaluating Healthy Islands programs (Ritchie *et al.*, 1998). Using this approach enabled an in-depth exploration of successful programs, with consideration of how the experiences of these programs may be applied to other health promotion programs in the Pacific.

The word ‘program’ is used here to describe discrete, planned activities to promote health in various Pacific Island countries and territories. However, this is not intended to perpetuate the myth that health promotion ends when funding runs out. We consider the end point for health promotion interventions to be when the changes sought have become embedded into the systems and structures of society to the extent that related health benefits are continued after the initial program funding ends (adapted from Shediac-Rizkallah and Bone 1998).

###  

#### Participants

Participants (*n* = 35) were those responsible for coordinating and/or supporting the four HIA awarded programs in 2013 and the four HIA awarded programs in 2015 (Table 1). The list of 2013 and 2015 winners of the award was used to identify the focal point/lead for the program, who was contacted to obtain informed consent for participation in this study. A snow-balling technique was used to identify other key informants for each program. Field visits to the location of six of the programs were undertaken by CB and EM in the 2015–2016 period. All participants were provided with a plain language statement about the study and provided written consent for de-identified information from their interviews to be published. Ethical approval was provided by Deakin University (HEAG-H 170_2015), and verbal approval was obtained from senior Ministry of Health staff in other countries.

**Table 1:** Healthy Islands recognition program awardees reviewed

Program Name	Location	Objective	Specific Actions	Lead Organisation	Year of Award
Let’s Go Local	Pohnpei, Federated States of Micronesia	Promoting locally-available foods using a variety of information, education and communication tools	Promoting local food consumption and production, maintaining gene bank of biodiversity, providing nutrition education	Island Food Community of Pohnpei	2013
Kau Mai Tonga	Tonga	Promote women’s participation in sport	Increase physical activity of women through promoting and providing netball facilities	Ministry of Internal Affairs	2013
Healthy Sianios and Samo Villages	Papua New Guinea	Develop a healthy village community	Participatory community action to remove still water, improve sanitation and beautify village setting	Ward 8 Development Committee, Lihir Island	2013
Honiara Central Market Healthy Setting Project	Solomon Islands	Create a healthy environment and promote sustainable food sources	Provision of improved sanitation, food preparation/storage and smoke-free environment in the Central Market	Honiara City Council & Ministry of Health	2013
PEN Fa’a Samoa	Samoa	Strengthen primary care for NCD prevention and control	A screening program to detect, manage and raise community awareness of NCDs	Ministry of Health, Samoa	2015
Scale up Sanitation Sanma Schools and Health Facilities	Sanma Province, Vanuatu	Improve sanitation in schools and health facilities	Development and construction of improved pit toilet design	Ministry of Health, Sanma Province	2015
Tasa Role Models	Commonwealth of the Northern Mariana Islands	Improving the health of children and families through role modelling	Establishment of role models to improve community facilities (parks, schools, etc.)	Northern Marianas College/TASA group	2015
Reducing Imports of Fizzy Drinks	Tokelau	Reducing sugar sweetened beverages	Policy advocacy for a ban on fizzy drinks	Ministry of Health, Tokelau	2015

### Data collection

In-depth, semi-structured interviews with key informants and review of internal and publicly available documentation were the techniques used to collect data. A simplified health promotion and evaluation cycle (situation analysis, planning, implementation and evaluation), was utilised as a framework for direction to appropriate documentation, guidance on the structure and nature of the questions asked in the interviews and to frame the discussion of results.

Interviews were audio-recorded, conducted in English (an official language in each of the countries/territories the programs were based in) and typically lasted 45 min to 1 h. Three telephone interviews were conducted where a visit was not possible, with questions sent to participants prior to the interview. Interviewers used questions drawn from the HIA application criteria. Participants were also asked to identify potential lessons for those interested in developing similar programs, including perspectives on challenges to effective health promotion in the Pacific and factors influencing sustainability.

To provide context and detail to the interview transcripts, documents were obtained from program staff, WHO and websites related to the HIA programs. Documents included HIA applications, organisational and program reports, news articles and website information.

### Analysis

Interview data were de-identified, transcribed and stored in an electronic database. Documentation data were collated and stored alongside the interview data. A thematic analysis of primary and secondary data sources was undertaken by CB and EM to describe successful programs within the context of each individual case, followed by a cross-case synthesis. In both of these stages, the simplified health promotion planning and evaluation framework was used to guide the analysis. A draft of the initial findings was prepared, and a workshop was held in June 2016 with, based on availability, representatives from three of the Healthy Islands Recognition Program Awardees. Findings were reviewed and discussed in this workshop. Separately, all participants were offered the opportunity to review and comment on findings to ensure accurate interpretation. Themes are discussed according to the health promotion planning and evaluation framework and illustrated using de-identified quotes.

## RESULTS

### Overview

Over 40 documents/websites were reviewed, and 35 interviews (20 women and 15 men) were undertaken over a 5-month period. Eight participants were program leads, while the remaining 27 were implementers and other stakeholders.

Program objectives (referred to in Table 1) targeted a range of modifiable risk factors for chronic disease including promotion of local foods (Pohnpei, Solomon Islands), restricting or controlling unhealthy foods (Tokelau), improving sanitation and controlling smoking (Solomon Islands) and promoting women’s physical activity (Tonga). They targeted vulnerable groups (e.g. children in the Commonwealth of the Northern Mariana Islands) or whole communities (e.g. villages in Papua New Guinea).

### Key challenges

While the HIA programs were recognised for their success, it is important to mention that they, like many health promotion programs, were faced with substantial challenges. These challenges were categorised under the broad themes of capacity, commitment and prioritisation, coordination and communication and other context-related challenges.

A lack of capacity, especially in terms of funding, resources and staffing, was the most common theme referred by participants and this affected the vast majority of programs.

*‘We really came to experience [an] obstacle because when [a key staff member was no longer able to work for the organisation], that was the end of it. We [weren’t] able find somebody who can work like [they] worked…it’s a big challenge and until now we don’t have a permanent replacement for [them]’.* (Interviewee A6)

In numerous cases, program and community need exceeded program capacity:

*‘There is high demand…but there is not the capacity to provide enough for everyone’.* (Interviewee E2)

As a result, some programs were reliant on short term sources of funding or contracts. Logistical challenges (particularly in rural and remote areas) also affected the reach of some programs. For example, it was very difficult for one program to reach outer islands and such communities were not able to fully benefit from its activities. Participants from one program also cited a particular need for further capacity building of their local volunteers and staff in health promotion and data collection.

Commitment to and prioritisation of the program amongst staff, the community and key stakeholders was another theme referred to. For example, one program was severely affected by a typhoon which reoriented community priorities to more immediate concerns during that period of time. Representatives of another program, which was reliant on the work of volunteers, mentioned that motivation from these volunteers ebbed and flowed over time. It was mentioned by participants from two programs that limited commitment from the government was a key challenge, and that initial government support may not necessarily be sustained.

In the theme of ‘coordination and communication’, challenges experienced by a small number of programs included maintaining the involvement of key stakeholders after program staff turnover, the need for better coordination between key stakeholders, and the need for improved information systems. For example, participants from one program cited the need to be able to conduct on-going surveys as a way to monitor the effectiveness of the program, which could also inform dialogue with the health department.

Some programs experienced other challenges that were unique to their context or specific health issue that they sought to address. For example, variations in access to health care, provision of water, and fresh local foods were occasionally an issue for programs that were dependent on these. In the case of Tokelau, where fizzy drinks were banned, the illicit trade of such beverages has been reported to be a potential challenge (McDonald, 2015).

### Key success factors and recommendations

Participants were asked how their programs became successful and how others could emulate this. Their responses are presented within the following stages of the simplified health promotion and evaluation cycle.

#### Situation analysis

Three broad themes were identified as contributing to effective situation analysis: building an evidence base; drawing on local practices, experiences and needs; and adapting global targets, issues, tools and approaches.

In most cases, programs assessed the current health situation and its causes by conducting desk-based reviews of scientific and other sources of information, and discussing and sharing this information with their communities. In Tokelau for example, evidence on the prevalence of overweight and obesity from a WHO STEPwise approach to Surveillance (STEPS) survey stimulated action on reducing sugar-sweetened beverages. This was supported by data collected by the Ministry of Health in Tokelau which found that soft drink consumption was approximately 43 L per person per annum (personal communication).

*‘When our people have the right information and understanding of their health status, the risk and appreciate where they want to go and the health they wish for, then they are empowered to make healthy decisions’.* (Interviewee G1)

This evidence was presented to the leaders (Taupulega), of each atoll. As a consequence, one atoll (Fakaofo) completely banned carbonated soft drinks in 2011. Local bans were subsequently introduced to Nukunonu and Atafu, and a national policy that banned imported fizzy drinks was introduced in 2013.

Participants recognised that while evidence is helpful for pointing them in the right direction, local knowledge was also critical for engaging the community and achieving shared responsibility. Local people often have the best understanding of community needs and offered creative solutions (WHO, 2017). Drawing on local culture and engaging the community was a particular strength of the Let’s Go Local program in Pohnpei, Federated States of Micronesia:

*‘[It’s important to] explain it in the context that it’s from the locally community’s perspective. It’s the reason why [the Island Food Community of Pohnpei] emphasise culture, as opposed to an economic perspective. They use that as an entry point to garner support from the community. In the [Island’s] culture, if you engage in their cultural activities and respect [it], they recognise you’re being serious…it gains your credibility and because of [that] it gives them a means to advocate on your behalf to other communities’.* (Interviewee A5)

Participants explained that linking with global targets, issues, tools and approaches gave them direct access to international best practice. Aspirational targets (e.g. the WHO’s (2014) target for no increase in childhood overweight by 2025) are useful for motivating behavioural and systems level change. PEN Fa’a Samoa adapted the WHO Package of Essential Noncommunicable Disease Interventions (PEN) so that communities owned the intervention and its delivery had a local flavour. Participants recognised that adapting such targets and guidelines to the local situation set them up well for the next step of planning.

#### Planning

Participants suggested that in the planning stage, information and partners should be brought together to identify, consider and prioritise possible solutions (WHO, 2017). For example, the Healthy Sianios and Samo Villages program brought together local villagers, representatives from interested organisations, and health professionals to plan interventions in a Community Action and Participation Training Program (an initiative of the Papua New Guinea National Department of Health). Several participants mentioned the importance of aligning these solutions strategically with existing resources (amount, type, source and focus) to maximise the chance of addressing the health issue sustainably. In this way, solutions are situated within the local environment and can be achieved, in many cases, with the resources and expertise at hand. The quote below, demonstrates how information, partners and resources were brought together to improve sanitation:

*‘[Our goal] was to get [students] to understand hygiene and also teach their parents. School and health committees with members from the community [were taught] how to build toilets so they can teach others…We brought in the Ministry of Education…to get data on basic needs for schools. They gave us names of schools in need of sanitation support’* (Interviewee E1)

Most participants considered a written plan essential for achieving a common understanding of the program goal and strategies (WHO, 2017). It was mentioned that plans need to be detailed enough for someone picking it up to understand and proceed with the program. Participants recommended that the plan clearly specify who is responsible for implementing specific strategies within it, along with when and how they will be implemented and how they will be resourced.

*‘A major learning for me was planning, planning, planning. We in the Pacific are talking people and we don’t write things down. But I realised how important it was to plan and record as you go along. I use the documents we developed now for other areas. I had more professional growth from being involved in the project than from doing a [university] degree’* (Interviewee B2)

They also recommended that the plan detail how the program will be evaluated to measure the overall success of the program but also to inform its delivery along the way. Finally, it was suggested that the ‘plan should be frequently updated, so that it can respond to new information, events, or changes to the health issue’ (WHO, 2017). Good planning, through ensuring sufficient time, budget, specifying clear objectives and having deadlines, was also seen as key to implementation (WHO, 2017).

#### Implementation

To implement a planned program, participants emphasised being realistic and flexible enough to make adjustments where necessary. For example, stakeholders involved with the Tasa Role Models program needed to adjust plans, timelines and budgets after a typhoon caused significant disruption. Another key to implementation was the reorientation of networks, communities and systems. Reorientation helped to embed the changes in the systems (such as the food system) and settings (such as retailers) so that they become the norm. Achieving reorientation required continuous communication, as evident in the Honiara Central Market Healthy Setting Project:

*‘One very important thing is collaboration with stakeholders and the partnership with other agencies that are involved…in the market itself, there are lots of issues to be addressed, so there have been meetings with other partners on how we can integrate into a working partnership to help develop the market…the [government health department] does the implementation, but they’ve had a series of meetings with other partners [that are involved in the management of the market]’.* (Interviewee H2)

Building the capacity and number of stakeholders was also key as this led to shared workloads, ownership, as well as expanding the reach of the program and resources. Finally, participants identified accountability as essential for effective implementation. A variety of accountability structures were evident in the programs including management and governance structures, reporting procedures and memorandums of understanding guiding how partners worked together. These structures were not necessarily started from scratch and, in fact, existing structures were used in many cases. The Kau Mai Tonga program provided a good example of implementation that was well planned and that had clear accountability.

*‘Project management mechanisms worked really well. It is hard to operationalise multisectoral interventions because organisations have different values. It worked in this project through:*

• *An overarching partnership agreement among key implementing partners*
• *Shared logic models*
• *Clear tasks for each organisation*
• *Aligning interests so that the supply side and the demand side were funded*
• *Shared monitoring and evaluation framework.*
• *Ministry of Internal Affairs would write up the work. This documentation of what we were doing created a shared view. Transparent with documentation, budgets, progress reports’.*

(Interviewee B3)

#### Evaluation

Participants pointed out the value of evaluation by describing it as telling their story. Some used logic models to help evaluate various stages of the program, including what worked and what didn’t work in terms of process, impacts and outcomes (WHO, 2017). Participants also noted that good evaluation helped keep funders, stakeholders and communities up to date with the program. Several programs were able to source and make good use of high quality research to inform the program and to demonstrate success. However, participants noted that large data sources and complex analysis were not always needed and may create more work than necessary.

A key observation was that program leaders did not wait until the ‘end’ of the implementation period before evaluation commenced. In fact, workshop participants recommended that evaluation is conducted from the beginning, as having baseline data prior to any proposed interventions was vital. For example, in the PEN Fa’a Samoa program, successes measured in pilot villages were important to support the program’s expansion to the national level. Representatives of the Kau Mai Tonga program tested social marketing advertisements in focus groups and reported back to stakeholders before the final version went to air so they could ensure they had messages that resonated with the target audience.

A formal, rigorous evaluation was noted by participants as potentially a resource-intensive and overwhelming task for smaller programs in the Pacific. For example, there was little evidence of the use of evaluation frameworks like RE-AIM. To overcome this, workshop participants emphasised to simply ‘start somewhere’ (i.e. on a small scale) and garner support from other stakeholders. Universities or non-government organisations can bring a wide range of technical expertise to evaluation and may also be able to attract additional resources. In starting on a small scale, the simple observation of children playing on playground equipment built as part of the Tasa Role Models program was used to demonstrate effectiveness. Another strategy was drawing on existing data. The Tokelau program used WHO STEPs data to justify the ban on sugar-sweetened beverages, and further iterations of WHO STEPs data can also be used to evaluate the impact. If existing surveys do not exist, the logistics, infrastructure and expertise of institutions that already run other surveys could still be utilised to either arrange for a new survey to add new questions/measurements to an existing survey.

## DISCUSSION

From document analysis and interviews with leaders and representatives of eight health promotion programs, we have described key challenges and success factors in the reviewed health promotion programs in the Pacific to inform best practice. These are summarised in Table 2.

**Table 2:** Summary of key challenges, success factors and recommendations in the HIA programs examined

Challenges	Success factors and recommendations
Limited capacity: Limited finances and sustainable funding Limited resources compared to demand Logistical challenges (particularly in rural and remote areas) Training needs	Community focus: Significant participation, involvement, ownership and engagement from the local community Drawing on local practices, experiences and needs Adapting global targets, issues, tools and approaches to the local situation

Commitment and prioritisation: Commitment and motivation ebbed and flowed amongst staff, the community and key stakeholders Government support may change or revert Disruption of program and community priorities due to external events	Collaboration: Engaging with and building the capacity of stakeholders to share workload, ownership and expand reach Ensuring clear governance and accountability structures

Communication and coordination: Staff turnover and maintaining involvement of key stakeholders Need for improved health information system with better communication and coordination	Sound planning and implementation: A clear, detailed, written plan that is updated Considering and prioritising possible solutions with program partners, and aligning these strategically with existing resources Being realistic, flexible and making adjustments where necessary Strategically aligning solutions with existing resources

Context-related challenges: Variations in access to services, facilities or goods that the program is dependent upon	Using evidence, evaluation and research: Building an evidence base to support situation analysis Pilot testing where necessary, prior to expansion Using logic models to support evaluation Starting on a small scale and/or using existing surveys and research infrastructure where necessary

Although framed within the Healthy Islands vision, the factors for success embedded within the programs examined align well with the existing (local and global) evidence on what makes for successful health promotion programs. Milat *et al.* (2015), in a review on success factors for scaling up public health interventions, highlighted several factors that align with this research, such as establishing monitoring and evaluation systems, financing models, active engagement of a range of implementers and the target community, tailoring approaches to the local context, systematic use of evidence, strong leadership and champions. Likewise, in obesity prevention, Whelan *et al.* (2018) identified resourcing, community engagement, partnerships, communication, adaptation and evaluation as critical for success and sustainability.

Amongst the programs examined in this study, the need for local community input, engagement, participation, leadership and ownership stood out as critical for success, supporting observations from various other studies in the Pacific region (Galea *et al.*, 2000; Litidamu (cited in Ireland *et al.*, 1996); Siefken *et al.*, 2012; Waqa *et al.*, 2013). This also resonates with the broader international literature on best practice health promotion, where community involvement is emphasised in key initiatives such as Healthy Cities (WHO, 2002) and the Ottawa Charter (WHO, 1986).

While there are similarities between what works in mainstream health promotion practice and the programs explored in this Pacific study, there are some differences. Capacity and limited resourcing constrain many health promotion programs globally (Milat *et al.*, 2015; Whelan et al. 2018) and there are also other examples in the Pacific (Schulenkorf and Siefken 2019) where such issues may be particularly acute. However, many of the programs reviewed here demonstrated that health promoters and communities in the Pacific can and do adapt to such constraints through creativity and innovation, and by being efficient and strategic. Capacity and resourcing constraints also magnify the importance of community involvement, collaboration and shared ownership to ensure the sustainability of health promotion programs, and consequently the Healthy Islands vision, in the Pacific.

### Strengths

A key strength of this study is the diverse range of programs and participants involved. The programs span various health issues, operated at different levels, were at different stages of the health promotion planning and evaluation cycle, and a wide range of Pacific Island countries and territories were included. Finally, the qualitative case study design enabled rich and detailed data directly from program leaders.

### Limitations

The methodology employed is exploratory. The case study design can also be challenged in terms of reliability and construct validity, although the adoption of recommended techniques such as using multiple sources of evidence, having draft reports reviewed by key informants, triangulation using multiple sources of data, and maintaining a database (Yin, 2014), helped to improve this. Programs were selected based on whether they received a WHO Healthy Island Recognition Award, which could introduce some bias as they may be in some way different to other successful programs in the region. Field visits to most sites in this study enabled the researchers to be immersed in the program’s settings, and interview most key informants in person. However, a challenge was that a small proportion of potential informants were away (or ‘off island’) during that particular time. In addition, researchers were not able to visit the physical location of programs in Samoa and Tokelau due to scheduling constraints. In both circumstances, telephone interviews were undertaken, and relevant documents were sent via email to the researchers. The health promotion planning and evaluation cycle was a useful lens through which to contextualise and explore how HIA program leaders successfully drove their respective programs, along with their recommendations for other health promotion practitioners in the Pacific. However, we used a simplified version and Pacific health promoters should draw on relevant evidence-based health promotion and evaluation frameworks wherever possible—the *Framework of Action for Revitalization of Healthy Islands in the Pacific* (WHO, 2013) incorporates many of these. It is also important to recognise that it should not be considered linearly. For example, to achieve the ‘full circle’ of the cycle, the evaluation should serve to inform its next iteration, and in particular, the objectives of the program should be revisited and one needs to consider if the target has been met. Furthermore, programs may evolve after repeated evaluations and shifts in their objectives and activities take place.

## CONCLUSION

This study identified and explored factors affecting the success of eight Healthy Islands award-winning health promotion programs in the Pacific. We found that using evidence, careful planning, building capacity, developing partnerships, strengthening and reorientating networks, ensuring accountability and conducting evaluation contributed to success. Recognising local settings and involving community (whether it be in terms of needs, adaptation, engagement, participation or leadership) was perhaps the most prominent theme. It is important to recognise that this study explored a selection of programs in the Pacific, however, it is far from all-encompassing and nor should all countries, territories, islands and communities of the Pacific be treated homogenously. Whilst this study adds to the scant amount of literature on health promotion on the Pacific, it is explorative and there is a vast need for further research in this area, which should allow for greater coverage of a wider range of health topics/issues and levels (i.e. village, city, island, national) across the various stages of the health promotion and evaluation cycle.

## FUNDING

This work was undertaken with funding support from the Government of Korea's Ministry of Health and Welfare through the World Health Organization's Division of Pacific Technical Support.

## Footnotes

1 This last point was added after the 1999 Palau Action Statement on Healthy Islands

## References

[daac002-B1] Galea G. , Powis B., Tamplin S. A. (2000) Healthy Islands in the Western Pacific—international settings development. Health Promotion International, 15, 169–178.

[daac002-B2] Ireland J. , Powis B., Litidamu N. (1996) Roots and wings of Healthy Islands: the history and potential of environmental health in the Pacific. Pacific Health Dialog, 3, 259–265.

[daac002-B3] McDonald A. (2015) Sugar-Sweetened Beverage Tax in Pacific Island Countries and Territories: A Discussion Paper. Secretariat of the Pacific Community, Noumea.

[daac002-B4] Milat A. J. , Bauman A., Redman S. (2015) Narrative review of models and success factors for scaling up public health interventions. Implementation Science, 10, 113. 26264351 10.1186/s13012-015-0301-6 PMC4533941

[daac002-B5] Ritchie J. , Rotem A., Hine B. (1998) Healthy Islands: from concept to practice. Pacific Health Dialog, 5, 180–186.

[daac002-B6] Schulenkorf N. , Siefken K. (2019) Managing sport-for-development and healthy lifestyles: the sport-for-health model. Sport Management Review, 22, 96–107.

[daac002-B7] Shediac-Rizkallah M. C. , Bone L. R. (1998) Planning for the sustainability of community-based health programs: conceptual frameworks and future directions for research, practice and policy. Health Education Research, 13, 87–108. 10178339 10.1093/her/13.1.87

[daac002-B8] Siefken K. , Macniven R., Schofield G., Bauman A., Waqanivalu T. (2012) A stocktake of physical activity programs in the Pacific Islands. Health Promotion International, 27, 197–201. 21561985 10.1093/heapro/dar026

[daac002-B9] Waqa G. , Moodie M., Schultz J., Swinburn B. (2013) Process evaluation of a community-based intervention program: healthy Youth Healthy Communities, an adolescent obesity prevention project in Fiji. Global Health Promotion, 20, 23–34. 24469301 10.1177/1757975913501909

[daac002-B10] Whelan J. , Love P., Millar L., Allender S., Bell C. (2018) Sustaining obesity prevention in communities: a systematic narrative synthesis review. Obesity Reviews, 19, 839–851. 29603583 10.1111/obr.12675

[daac002-B11] WHO (1986) Ottawa Charter for Health Promotion: First International Conference on Health Promotion, Ottawa, 21 November 1986. https://www.who.int/healthpromotion/conferences/previous/ottawa/en/ (last accessed 25 November 2020).

[daac002-B12] WHO (1995) Yanuca Islands Declaration on Health in the Pacific in the 21st Century: Meeting of the Ministers of Health for the Pacific Island Countries. WHO, Yanuca Island, Fiji.

[daac002-B13] WHO (1998) Health Promotion Glossary. WHO, Geneva.

[daac002-B14] WHO (2002) Community Participation in Local Health and Sustainable Development: Approaches and Techniques. WHO Regional Office for Europe, Copenhagen.

[daac002-B15] WHO (2013) Framework of Action for Revitalization of Healthy Islands in the Pacific. WHO, Geneva.

[daac002-B16] WHO (2014) Global Nutrition Targets 2025: Policy Brief Series (WHO/NMH/NHD/14.2). WHO, Geneva.

[daac002-B17] WHO (2015) The First 20 Years of the Journey towards the Vision of Healthy Islands in the Pacific. WHO, Geneva.

[daac002-B18] WHO (2017) Healthy Islands: Best Practices in Health Promotion in the Pacific. Licence: CC BY-NC-SA 3.0 IGO.

[daac002-B19] Yin R. K. (2014) Case Study Research: Design and Methods. Sage Publications, Thousand Oaks, CA.

